# General Hospital for the University of Pennsylvania

**Published:** 1873-01

**Authors:** William Pepper

**Affiliations:** Chairman of Finance Committee.; Philadelphia, 1215 Walnut Street


### GENERAL HOSPITAL FOR THE UNIVERSITY OF PENNSYLVANIA.

We take pleasure in publishing and calling attention to the following circular, which gives information in regard to the proposed establishment of a General Hospital for the University of Pennsylvania, of which institution the Senior Editor of this journal is an alumnus, and in the perpetuation of its commanding influence and facilities for medical education, feels the deepest interest. It is hoped that all the alumni of the institution, wherever found or however much depressed in circumstances will be pleased to have the opportunity of contributing something toward the combined usefulness and prosperity of their *alma mater.*

PHILADELPHIA, 1215 Walnut Street.

DEAR DOCTOR,—A little more than a year ago the movement was inaugurated for the establishment of a General Hospital in connection with the University of Pennsylvania. The reasons which have led to this important step are obvious. For some years past the number of applicants for medical and surgical aid at the University has been rapidly increasing, until, during the past year, nearly 5000 patients have been treated in the various departments of the Dispensary. A large proportion of these cases, however, are always of such a character as to require admission into a Hospital Ward for proper treatment. The restricted space hitherto at the disposal of the Faculty has entirely prevented them from affording this much-needed shelter. Not only this, but the vast growth of population, especially of the artisan classes in Philadelphia, and the unprecedented development of the mining and manufacturing interests of the State at large, have caused such increased demand for Hospital accommodation, that scarce a day passes without numbers of worthy applicants being rejected from the doors of each of our Hospitals.

The desire of affording increased clinical facilities to the students of the University was another very potent argument in favor of this enterprise. The Faculty have uniformly striven to afford the largest possible amount of bedside study of medical and surgical disease; but notwithstanding the advantages furnished by the public Hospital Clinics, and those of the University Dispensary, experience has shown the necessity of a General Hospital under the immediate direction of the School. The advantages of such a union, both to patients and students, are evident. The reputation of the School will largely depend upon the model character of the Hospital, and it is therefore certain that the highest obtainable technical skill will be directed, not only by educated benevolence, but also by self-interest, to develop the greatest efficiency in the cure of disease, and the material comfort of the patients. The advantages to medical education will also be great. The students of the University will have daily access to the Hospital Wards, and will have every facility for acquiring a thorough practical knowledge of their profession. A large number of positions, as Resident Physicians, Assistants, and Dressers, will also be thrown open to members of the University class, and will secure to the successful aspirants unrivalled advantages.

In order to fully meet the wants of the community, the Trustees have decided upon the erection of a Hospital which shall contain at least 200 beds. The city of Philadelphia has, with wise liberality, given as a building site, the square, comprising five and a half acres, embraced between Thirty-fifth and Thirty-sixth streets, and Spruce and Pine streets. The cost of building and endowing such a Hospital will be at least $500,000. Towards this amount, the State of Pennsylvania has appropriated $100,000 for building purposes: a munificent individual, Mr. I. V. Williamson, has contributed $50,000; and the additional subscriptions amount to $210,000 (*see* enclosed list). The plans have been finally adopted, after prolonged and careful preparation, and the work on the buildings has already begun.

At a meeting of the Alumni of the Medical Department of the University, held in May, 1872, it was unanimously resolved, as the most fitting and permanent expression of interest in this noble cause, to raise a fund of at least $50,000 for the endowment of an "Alumni Ward" in this Hospital. A list of the subscriptions already made to this special fund is enclosed. You are cordailly invited to subscribe to this object, and to exert your influence with other alumni in furthering its success.

Subscriptions should be sent to Dr. JAMES TYSON, 322 South Fifteenth street, Philadelphia, the Treasurer of this special fund, and may be made payable immediately, or in four annual instalments, at the option of the donor.

Very truly yours, WILLIAM PEPPER,
*Chairman of Finance Committee.*

OCTOBET 15, 1872.

